# A Low or High Physical Activity Level Does Not Modulate Prostate Tumor Tissue Protein Synthesis Rates

**DOI:** 10.1249/MSS.0000000000003349

**Published:** 2023-11-27

**Authors:** LISANNE H. P. HOUBEN, TOM TUYTTEN, ANDREW M. HOLWERDA, ERIKA WISANTO, JOAN SENDEN, WILL K. W. H. WODZIG, STEVEN W. M. OLDE DAMINK, MILOU BEELEN, SANDRA BEIJER, KOENRAAD VAN RENTERGHEM, LUC J. C. VAN LOON

**Affiliations:** 1 Department of Human Biology, NUTRIM School of Nutrition and Translational Research in Metabolism, Maastricht University Medical Centre+, Maastricht, THE NETHERLANDS; 2 Department of Research & Development, Netherlands Comprehensive Cancer Organisation (IKNL), Utrecht, THE NETHERLANDS; 3 TiFN, Wageningen, THE NETHERLANDS; 4 Department of Urology, Jessa Hospital, Hasselt, BELGIUM; 5 Department of Pathology, Jessa Hospital, Hasselt, BELGIUM; 6 Central Diagnostic Laboratory, Maastricht University Medical Centre+, Maastricht, THE NETHERLANDS; 7 Department of Surgery, NUTRIM School of Nutrition and Translational Research in Metabolism, Maastricht University Medical Centre+, Maastricht, THE NETHERLANDS; 8 Department of Visceral and Transplantation Surgery, RWTH Aachen University, Aachen, GERMANY; 9 Department of Dietetics, Maastricht University Medical Centre+, Maastricht, THE NETHERLANDS; 10 Faculty of Medicine, Hasselt University, Hasselt, BELGIUM; 11 Department of Urology, University Hospital Leuven, Leuven, BELGIUM

**Keywords:** EXERCISE, PROSTATE CANCER, TUMOR GROWTH, TUMOR METABOLISM, SKELETAL MUSCLE

## Abstract

**Introduction:**

Physical activity level has been identified as an important factor in the development and progression of various types of cancer. In this study, we determined the impact of a low versus high physical activity level on skeletal muscle, healthy prostate, and prostate tumor protein synthesis rates *in vivo* in prostate cancer patients.

**Methods:**

Thirty prostate cancer patients (age, 66 ± 5 yr; body mass index, 27.4 ± 2.9 kg·m^−2^) were randomized to a low (<4000 steps per day, *n* = 15) or high (>14,000 steps per day, *n* = 15) physical activity level for 7 d before their scheduled radical prostatectomy. Daily deuterium oxide administration was combined with the collection of plasma, skeletal muscle, nontumorous prostate, and prostate tumor tissue during the surgical procedure to determine tissue protein synthesis rates throughout the intervention period.

**Results:**

Daily step counts averaged 3610 ± 878 and 17,589 ± 4680 steps in patients subjected to the low and high physical activity levels, respectively (*P* < 0.001). No differences were observed between tissue protein synthesis rates of skeletal muscle, healthy prostate, or prostate tumor between the low (1.47% ± 0.21%, 2.74% ± 0.70%, and 4.76% ± 1.23% per day, respectively) and high (1.42% ± 0.16%, 2.64% ± 0.58%, and 4.72% ± 0.80% per day, respectively) physical activity group (all *P* > 0.4). Tissue protein synthesis rates were nearly twofold higher in prostate tumor compared with nontumorous prostate tissue.

**Conclusions:**

A short-term high or low physical activity level does not modulate prostate or prostate tumor protein synthesis rates *in vivo* in prostate cancer patients. More studies on the impact of physical activity level on tumor protein synthesis rates and tumor progression are warranted to understand the potential impact of lifestyle interventions in the prevention and treatment of cancer.

Prostate cancer is the second most common cancer and the fifth leading cause of death in men worldwide (1). Lifestyle modifications are rapidly becoming recognized as important adjunct therapeutic approaches to slow cancer development and enhance treatment efficacy. Epidemiological data suggest that physical activity protects against the development and/or progression of several types of cancer (2,3), including prostate cancer (4–11). Furthermore, emerging preclinical evidence in animal models indicates that increased physical activity (e.g., wheel running, swimming) can strongly reduce tumor growth (12,13).

Tumor growth is regulated by the balance between tumor protein synthesis and breakdown rates. Evidence suggests that higher physical activity levels downregulate translational signaling involved in the regulation of tumor protein synthesis, with enhanced AMPK activity and suppressed mTOR activity appearing to play central roles (14–16). Several physical activity–mediated mechanisms have been proposed to elicit tumor growth-inhibiting effects, including modulation of hormonal/growth factors (e.g., insulin/insulin growth factor, testosterone) (17), release of myokines (12), improved immune function (17), and changes in tumor vascularization (18). However, although compelling preclinical data suggest that an increased physical activity level reduces tumor growth, establishing a therapeutic effect of increased physical activity level *in vivo* in humans has proven challenging because of the limited specificity of indirect biomarkers of tumor growth (e.g., prostate-specific antigen, Ki67) and the difficulty in accurately assessing tumor growth over prolonged intervention periods (19–21).

Recently, we applied perioperative intravenous stable isotope amino acid infusions with tumor tissue sample collection during resection surgery in pancreatic cancer patients as a means to quantify pancreatic tissue and pancreatic tumor tissue protein synthesis rates (22). This approach allowed us to assess tumor tissue protein synthesis rates over a 4- to 8-h timespan before resection. Although this provides us with important insight in the dynamics of tumor protein metabolism, it precludes the assessment of potential tumor-growth inhibiting effects to lower tumor protein synthesis rates. The recent reintroduction of the use of deuterium oxide (^2^H_2_O) administration provides a stable isotope tracer labeling methodology that allows us to assess tissue protein synthesis rates over a more extended period of several days or even weeks (23). Such expanded assessment periods provide us with the opportunity to assess the impact of lifestyle interventions on tissue protein synthesis rates. So far, this approach has been successfully applied to assess the impact of more (23) or less (24,25) physical activity on muscle tissue protein synthesis rates. To date, this approach has not been applied to assess the impact of physical activity level on tumor protein synthesis rates *in vivo* in (prostate) cancer patients.

In the present study, 30 prostate cancer patients scheduled for radical prostatectomy were subjected to either a low or high level of daily physical activity. In the week before surgery, patients performed either less than 4000 steps per day (low physical activity) or more than 14,000 steps per day (high physical activity). Deuterium oxide was provided throughout the intervention period with saliva, blood, muscle, nontumorous prostate and prostate tumor tissue sampling to allow subsequent assessment of skeletal muscle, healthy prostate, and prostate tumor protein synthesis rates. This methodology enabled us to directly assess the impact of a low versus high physical activity level on muscle and prostate (tumor) tissue protein synthesis rates *in vivo* in prostate cancer patients under free-living conditions. We hypothesized that a high physical activity level will result in higher protein synthesis rates in skeletal muscle and lower protein synthesis rates in healthy prostate and prostate tumor tissue when compared with a low physical activity level.

## METHODS

### Participants and ethical approval

A total of 30 prostate cancer patients were recruited to participate in the present study between June 2020 and June 2021. Patients were eligible for participation when they were scheduled for a robot-assisted radical prostatectomy with curative intent because of prostate cancer. Patients needed to be capable to perform walking activities required to participate in the physical activity intervention program. Patients with peripheral artery disease Fontaine III or IV, or with neuromuscular disorders or other comorbidities that seriously hampered mobility, were excluded. Potential subjects were identified by the urologist at the outpatient clinic of the Department of Urology of the Jessa Hospital, Hasselt, Belgium. All patients were informed of the nature and potential risks of the experimental procedure before informed written consent was obtained. The study was approved by the Ethical Review Committee (Ethische Toetsingscommissie) Jessa Hospital, Hasselt, Belgium (reference: B243202042677) and conformed to standards for the use of human participants in research as outlined in the Declaration of Helsinki. All patients provided written informed consent before participating in this study. The study was registered at Netherlands Trial Register as Trial NL8768.

### Study design

**FIGURE 1:**
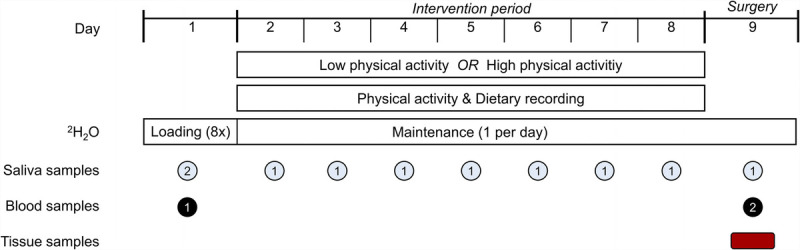
Graphic overview of the study design. Tissue samples of vastus lateralis muscle, healthy prostate, and prostate tumor tissue were collected ^2^H_2_O, deuterated water.

This study was a two-armed, randomized controlled trial. A graphic overview of the study design is presented in Figure 1. The study protocol consisted of 9 consecutive days. The first experimental procedures were conducted on the morning of day 1 at the patients’ homes. After obtaining informed consent, a fasting serum and saliva sample were collected, body weight was measured, and the deuterium oxide (^2^H_2_O) dosing protocol was initiated. Patients were then randomized to perform either the low (*n* = 15) or high (*n* = 15) physical activity intervention. The randomization was performed by an independent researcher who used computer-generated random numbers in permuted blocks of 6.

The intervention period ran from day 2 to day 8. During this period, patients in the low physical activity group were instructed to perform no more than 4000 steps per day, and patients in the high physical activity group were instructed to perform 14,000 steps or more per day. Other physical activities (e.g., cycling) were allowed in the high physical activity group if they were performed in addition to the 14,000 steps a day. Patients in the low physical activity group refrained from other physical activities besides the limited step count. Patients tracked their physical activity throughout the intervention period and recorded their food intake for 3 d. The researchers had daily telephone contact with the patients to provide constant guidance and ensure compliance to the ^2^H_2_O dosing protocol. During surgery, blood, vastus lateralis muscle, nontumorous prostate, and prostate tumor tissue samples were collected.

### Physical activity

Patients were instructed to refrain from strenuous exercise 48 h before the start of the study. On the first experimental day, patients were provided with an accelerometer, a physical activity diary, and a pedometer. The accelerometer (Actigraph wGT3X-BT; ActiGraph, Pensacola, FL) and physical activity diary were used to objectively track all physical activities during the intervention period (26,27). From day 2 to day 8, patients continuously wore the accelerometer during waking hours on the right hip. In addition, patients simultaneously tracked their physical activities in a 7-d physical activity diary. The pedometer (Yamax Digi-Walker SW-200; Yamasa Tokei Keiki Co. Ltd., Tokyo, Japan) was worn on the left hip during waking hours and was used to provide patients with real-time feedback on their step count. The Yamax Digi-Walker SW-200 is an often used pedometer in (clinical) studies (24,28–33), which provides a valid and reliable step count and performs well during a range of walking speeds (30,34–36). Before use, each pedometer was checked for accuracy by walking a short distance at normal walking pace and simultaneously counting the actual steps taken (37). Each evening before sleep, the displayed daily step count was recorded by the patient.

### Dietary intake

Patients were instructed to maintain their habitual diet as consistently as possible in the 48 h before the start of the study. During the study period, no dietary restrictions were imposed. Dietary intake was monitored by a 3-d food diary, filled in by the patient on two weekdays and one weekend day. Food diaries were analyzed for average energy and macronutrients intake using web-based software (Eetmeter; Voedingscentrum, Den Haag, The Netherlands).

### Deuterated water-dosing protocol

The ^2^H_2_O-dosing protocol consisted of 1 loading day and 8 maintenance days. The dosing protocol was identical to previously published studies that administered ^2^H_2_O in human participants (23–25,38,39). On day 1 (loading day), one background serum and one saliva sample were collected after an overnight fast. Thereafter, patients ingested 8 × 50 mL boluses of 70% ^2^H_2_O (Cambridge Isotopes Laboratories, Andover, MA) separated by 1.5 h to minimize the risk of side effects (e.g., dizziness or vertigo). Although 10 patients reported light feelings of dizziness possibly related to the loading protocol, this was very mild and not disabling, and had completely disappeared the next day. After completing the loading protocol, a second saliva sample was collected in the course of the evening. From day 2 to day 8, patients consumed one ^2^H_2_O dose of 50 mL each morning upon waking. Each evening, patients collected a saliva sample at least 30 min after consuming dinner or evening snack. During surgery, an arterial serum sample was taken at the moment of the actual prostate resection.

### Blood, saliva, and tissue collection and processing

Blood samples were collected in evacuated tubes (ST II Advance Tube; BD Vacutainer, Plymouth, United Kingdom) and allowed to coagulate for at least 90 min before centrifugation at 1000*g* for 15 min at 21°C. Aliquots of serum were frozen in liquid nitrogen and stored at −80°C. Saliva samples were collected using cotton dental swabs (Celluron, Hartmann, Germany). Patients lightly chewed on a cotton swab until saturated with saliva (at least 2 min). The swab was then removed and refrigerated in a sealed tube until collection on day 9. Saliva was extracted, frozen in liquid nitrogen, and stored at −80°C until subsequent analyses.

**FIGURE 2:**
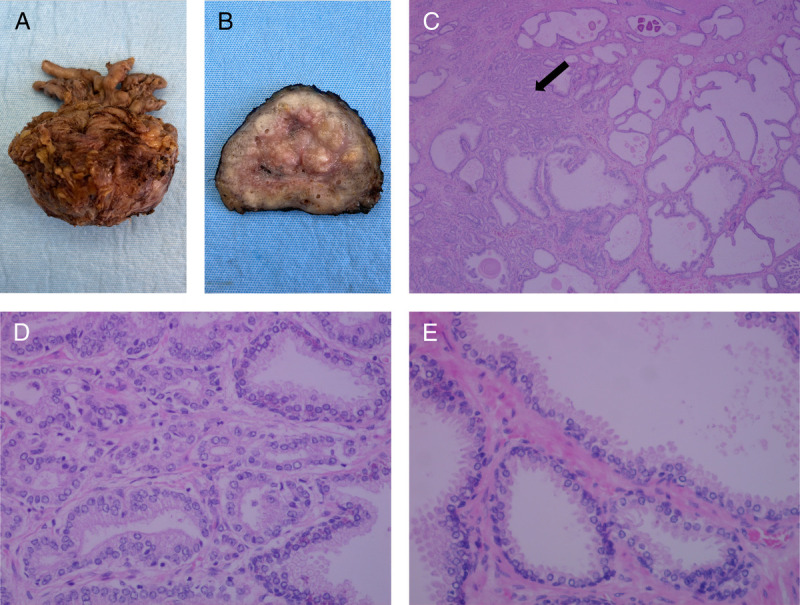
Prostate tumor biopsy. A, Macroscopy of the prostatectomy specimen. B, Macroscopy of cross section of prostatectomy specimen. C, Microscopy of coupe stained with HE, confirming the presence of adenocarcinoma (left, arrow) and healthy tissue (right), two times enlarged. D, Microscopy-confirming adenocarcinoma, 20 times enlarged. E, Microscopy-confirming healthy prostate tissue, 20 times enlarged.

Muscle, healthy prostate, and prostate tumor tissue sampling took place during the surgical procedure. The muscle biopsy was obtained under general anesthesia before the start of the prostatectomy procedure. The muscle biopsy was obtained from the middle region of the vastus lateralis muscle (about 15 cm above the patella) and about 3 cm below entry through the fascia, using a modified Bergström needle (40) with manual suction. Muscle biopsy samples were dissected carefully and freed from any visible nonmuscle material before being frozen in liquid nitrogen and stored at −80°C until further analysis. During the surgery, the prostate was resected, immediately fixed in formaldehyde 4%, and transferred to the pathology laboratory. The prostatectomy resection specimen tissue was subsequently macroscopically evaluated and further handled in accordance with the regular pathological procedures. Specifically, the formalin-fixed and paraffin-embedded tissue was cut into slices and stained with hematoxylin and eosin (HE), and carefully examined by the pathologist using light microscopy. When examination of the HE-stained slices was not fully conclusive, immunohistochemical stains (e.g., AMACR/P504S, p63, or 34BE12) were applied. Tumorous samples were characterized by the pathologist, including Gleason score and International Society of Urological Pathology (ISUP) gradation. Subsequently, samples of confirmed nontumorous prostate and viable prostate tumor tissue without signs of necrosis or ischemia were collected for further stable isotope analyses (Fig. 2).

### Serum hormones

Testosterone, C-reactive protein, insulin, and C-peptide were assessed in serum samples collected in a resting and fasting state before and after the intervention. Serum testosterone was measured by using an electrochemiluminescent immunoassay (Cobas 8000 instrument; Roche Diagnostics). Plasma C-reactive protein concentrations were measured using immunoturbidimetric assays (Cobas 8000 instrument; Roche Diagnostics). Insulin and C-peptide were measured by use of an Electric-Chemiluminiscence Immuno Assay on an Immulite XPi (Siemens Healthcare Diagnostics).

### Body water deuterium enrichments

Body water enrichment was analyzed using the saliva samples collected throughout the experimental protocol as described previously (23). Briefly, samples were diluted 70-fold and reacted in sealed vials to undergo deuterium equilibration with hydrogen gas. The deuterium enrichment of the hydrogen gas was then measured in duplicate by isotope ratio mass spectrometry (IRMS; DELTA V Advantage IRMS fitted with a GasBench II system and PAL system auto injector; Thermo Fisher Scientific, Bremen, Germany). Standard regression curves were applied from a series of known standard enrichment values against the measured values to assess the linearity of the mass spectrometer and to account for deuterium loss during equilibration.

### Serum-free [^2^H]alanine enrichments

Serum-free [^2^H]alanine enrichments were determined by gas chromatography–mass spectrometry analyses, as described previously (23). Briefly, serum samples were deproteinized and purified before free amino acids were converted into *tert*-butyldimethylsilyl derivatives with N-tert-butyldimethylsilyl-N-methyltrifluoroacetamide before analysis by gas chromatography–mass spectrometry (Agilent 5975C MSD and 7890A GC, Wilmington, DE). The plasma-free alanine mass isotopomers (M and M + 1) were determined using selective ion monitoring at *m*/*z* 232 and 233. Standard regression curves were applied from a series of known standard enrichment values against the measured values to assess the linearity of the mass spectrometer and to account for any isotope fractionation.

### Muscle, prostate, and prostate tumor tissue protein-bound [^2^H]alanine enrichments

For measurement of muscle protein-bound [^2^H]alanine enrichments, 30–60 mg wet muscle tissue was freeze dried. Collagen, blood, and other nonmuscle fiber material were removed from the muscle fibers under a light microscope. The isolated muscle fiber mass (5–10 mg) was weighed, and 35 volumes (35 times dry weight of isolated muscle fibers wet/dry ratio) of ice-cold 2% perchloric acid were added. The tissue was then sonicated and centrifuged. The protein pellet was washed with three additional 1.5-mL washes of 2% perchloric acid, dried, and hydrolyzed in 6 M HCl at 120°C for 15–18 h.

For measurement of [^2^H]alanine enrichment in healthy prostate and prostate tumor tissue proteins, visible paraffin was carefully removed from the samples. The isolated healthy prostate and prostate tumor tissue was weighed and 30–60 g was hydrolyzed in 6 M HCl at 120°C for 15–18 h. After hydrolyzation muscle, healthy prostate, and prostate tumor samples were dried under a nitrogen stream while being heated at 120°C. Samples were then dissolved with a 25% acetic acid solution before being passed over Dowex exchange resin (AG 50 W-X8, 100–200 mesh hydrogen form; Bio-Rad, Hercules, CA) by using 2 M NH_4_OH. Thereafter, the eluate was divided into two screw cap tubes and dried under a nitrogen stream for 24–36 h, and the purified amino acids were derivatized to their *N*(*O*,*S*)-ethoxycarbonyl ethyl esters (41). The derivatized samples were measured using a gas chromatography–isotope ratio mass spectrometer (MAT 253+; Thermo Fisher Scientific) equipped with a pyrolysis oven using a 60-m DB-17MS column and 5-m precolumn (No. 122-4762; Agilent) and GC-Isolink. Ion masses 2 and 3 were monitored to determine the ^2^H/^1^H ratios of muscle protein-bound alanine. A series of known standards were applied to assess linearity of the mass spectrometer and to control for the loss of tracer.

### Calculations

Tissue protein synthesis rates were calculated as fractional synthetic rate (FSR), expressed as %·d^−1^. FSR was determined using the incorporation of [^2^H]alanine into tissue proteins and the mean body water deuterium enrichment corrected by a factor of 3.7 based on the deuterium labeling during *de novo* alanine synthesis. Second, we repeated the calculations using mean free [^2^H]alanine enrichment in arterial serum instead of body water enrichment. The standard precursor-product method was used to calculate FSR:


$$\mathrm{FSR}\left(\%\cdot d^{-1}\right)=\left(\frac{E_{p2}-E_{p1}}{E_{\text{precursor}}\times t}\right)\times 100\%$$

where *E*_p1_ and *E*_p2_ are the protein-bound enrichments measured in the basal mixed serum protein and tissue samples (muscle, healthy prostate, prostate tumor tissue), respectively (single biopsy approach). *E*_precursor_ represents mean body water deuterium enrichment corrected by a factor 3.7, or mean free [^2^H]alanine enrichment in serum. *t* represents the ^2^H_2_O incorporation time.

### Statistical analyses

All data are expressed as means ± SD or as frequency and percentages. Baseline characteristics were compared between groups using independent samples *t*-tests (for continuous variables) or χ^2^ tests (for categorical variables). Physical activity and dietary intake data were compared between groups using independent-samples *t*-tests. Internal consistency between accelerometer and pedometer-derived average step count was assessed with Cronbach *α*. Serum hormone concentrations were compared using repeated-measures ANOVAs with time as within-subject and treatment as between-subject variables. Tissue-specific protein synthesis rates, expressed as fractional synthesis rates (FSR, %·d^−1^), were compared between groups using independent-samples *t*-tests for vastus lateralis muscle samples, prostate tissue, and prostate tumor tissue. For explorative purposes, FSR values were compared between the different tissues using repeated-measures ANOVA with tissue as within-subject factor and group as between-subject factor. All data were normally distributed, as assessed by Shapiro–Wilk test (*P* > 0.05) or visual inspection of the histograms. The only violation of normality was found for fat intake (percentage of energy) in the low physical activity group. As independent-samples *t*-tests are fairly robust to deviations from normality and to be consistent with the other comparisons, the independent samples *t*-test was run regardless. Statistical significance was set at *P* < 0.05. All analyses were performed using IBM SPSS Statistics (version 27.0; IBM Corp., Armonk, NY).

## RESULTS

Thirty-one prostate cancer patients scheduled for a radical prostatectomy with curative intent because of proven localized intermediate- to high-risk prostate cancer were included in the study, with 30 patients completing the study. In one patient, surgery was canceled 1 d before planning because of a possible COVID-19 infection. As a consequence, no tissues could be collected, and therefore, this patient was excluded from the analyses. To end up with groups of equal numbers, one additional patient was recruited (see Supplemental Fig. 1, Supplemental Digital Content, showing the CONSORT flow diagram, http://links.lww.com/MSSE/C951). Patient characteristics are presented in Table 1. Patients were, on average, 66 ± 5 yr old and were slightly overweight (body mass index, 27.4 ± 2.9 kg·m^−2^). None of the patients received neoadjuvant therapy. All prostate tumors were histopathologically classified as adenocarcinomas with 90% graded as ISUP 2 or 3. No differences were observed between the low and high physical activity groups in any of the patients’ characteristics (all *P* > 0.05).

**TABLE 1:** Patients’ characteristics.

	Low Physical Activity (*n* = 15)	High Physical Activity (*n* = 15)
Age (yr)	67 ± 6	66 ± 4
Height (m)	1.76 ± 0.06	1.77 ± 0.06
Weight (kg)	85.7 ± 13.0	85.2 ± 8.0
BMI (kg·m^−2^)	27.5 ± 3.3	27.3 ± 2.7
PSA, preoperative	7.2 ± 2.4	8.3 ± 4.0
Histopathological diagnosis		
Adenocarcinoma	30 (100)	30 (100)
Other	0 (0)	0 (0)
Gleason score		
7	14 (93.3)	13 (86.7)
8	0 (0.0)	1 (6.7)
9	1 (6.7)	1 (6.7)
Grade group (ISUP)		
2	8 (53.3)	8 (53.3)
3	6 (40.0)	5 (33.3)
4	0 (0.0)	1 (6.7)
5	1 (6.7)	1 (6.7)

Values are means ± SD, or number and (%) of patients. Data were compared between groups using independent-samples *t*-tests. No significant differences in patients’ characteristics were observed between groups.

BMI, body mass index; PSA, prostate-specific antigen.

### Physical activity

**FIGURE 3:**
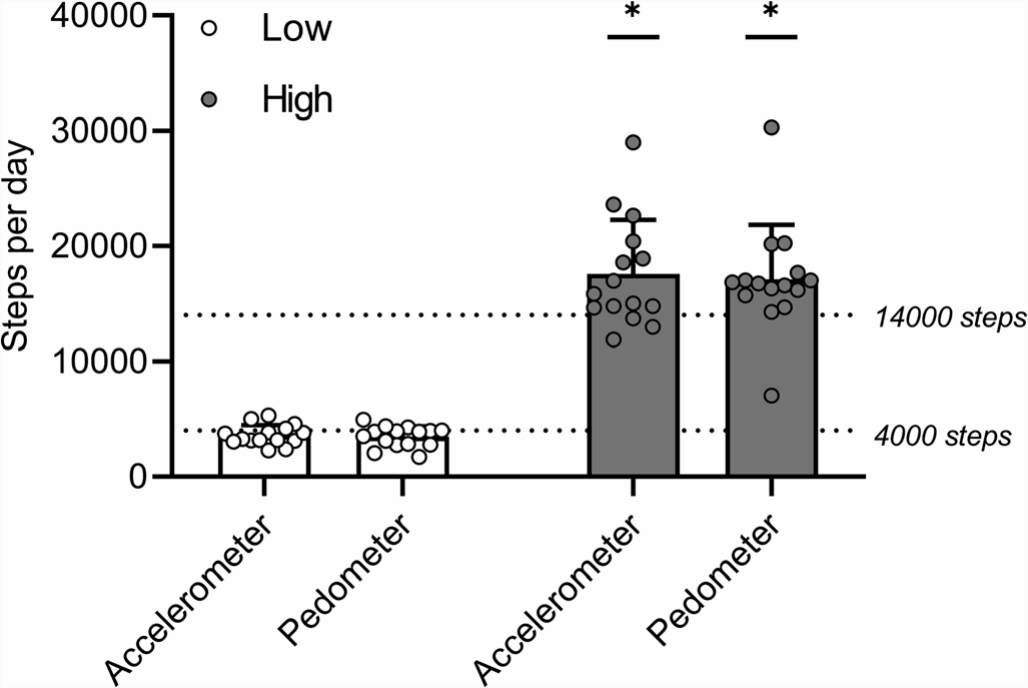
Daily step count as derived with the accelerometers and with the pedometers. Data were compared between groups using independent-samples *t*-tests. *Significantly different from daily step count in the low habitual physical activity group. Low, low physical activity group; High, high physical activity group.

The physical activity intervention was successful (Fig. 3), with an average daily step count of 3610 ± 878 and 17,589 ± 4680 performed in the low and high physical activity group, respectively (*P* < 0.001). Self-reported pedometer-derived step count was 3477 ± 905 and 17,127 ± 4725 steps per day, respectively (*P* < 0.001), and strongly correlated with the accelerometer-derived data (Cronbach *α* = 0.972). Absolute (in minutes per day) and relative (in percent) sedentary times were lower in the high versus low physical activity group (Table 2, *P* < 0.05). Absolute (in minutes per day) and relative (in percent) light, moderate, and vigorous physical activity times and moderate–vigorous physical activity were greater in the high versus low physical activity group (Table 2, all *P* < 0.05).

**TABLE 2:** Interventional physical activity.

	Low Physical Activity (*n* = 15)	High Physical Activity (*n* = 15)
Sedentary time (min·d^−1^)	684 ± 115	572 ± 78*^a^*
Sedentary time (%)	81 ± 5	65 ± 6*^a^*
Light activity time (min·d^−1^)	134 ± 15	174 ± 34*^a^*
Light activity time (%)	16 ± 4	20 ± 4*^a^*
Moderate activity time (min·d^−1^)	17 ± 9	132 ± 37*^a^*
Moderate activity time (%)	2 ± 1	15 ± 5*^a^*
Vigorous activity time (min·d^−1^)	0 ± 0	1 ± 1
Vigorous activity time (%)	0 ± 0	0 ± 0
MVPA time (min·d^−1^)	18 ± 9	133 ± 37*^a^*
MVPA time (%)	2 ± 1	15 ± 5*^a^*

Values are mean ± SD. Data were compared between groups using independent-samples *t*-tests.

*^a^*Statistically significant from low physical activity group (*P* < 0.05).

MVPA, moderate–vigorous physical activity.

### Dietary intake

Dietary intake data during the intervention are presented in Table 3. Because of apparent underreporting, the data from one patient were excluded from analyses. Energy intake was significantly higher in the high compared with the low physical activity group (10.7 ± 1.6 and 9.1 ± 1.4 MJ·d^−1^, respectively; *P* = 0.008). Energy intake was for 17% ± 3% provided by protein, 41% ± 9% by carbohydrate, and 36% ± 6% by fat, with no differences between groups. Daily protein intake was not different between groups and averaged 1.1 ± 0.3 and 1.2 ± 0.3 g·kg body weight·d^−1^ in the low and high physical activity groups, respectively.

**TABLE 3:** Interventional dietary intake.

	Without Outlier				All Patients				Between Group Differences (*P*-Value)	
	*n*	Mean	±	SD	*n*	Mean	±	SD	Without Outlier	All Patients
Energy intake (MJ·day^−1^)									0.008	0.078
Low	15	9.1	±	1.4	15	9.1	±	1.4		
High	14	10.7	±	1.6	15	10.3	±	2.2		
Protein intake (g·kg BW^−1^·day^−1^)									0.285	0.461
Low	15	1.1	±	0.3	15	1.1	±	0.3		
High	14	1.2	±	0.3	15	1.2	±	0.4		
Protein intake (% of energy)									0.502	0.527
Low	15	17.4	±	3.3	15	17.4	±	3.3		
High	14	16.5	±	3.3	15	16.6	±	3.2		
Carbohydrate intake (% of energy)									0.081	0.062
Low	15	43.4	±	8.3	15	43.4	±	8.3		
High	14	37.6	±	8.9	15	37.4	±	8.6		
Fat intake (% of energy)									0.109	0.081
Low	15	34.4	±	7.7	15	34.4	±	7.7		
High	14	38.2	±	4.0	15	38.5	±	4.1		

Values are mean ± SD. Data were compared between groups using independent samples t-tests. BW, body weight; Low, low physical activity group; High, high physical activity group.

### Serum hormone concentrations

Serum hormone concentrations of testosterone, insulin, CRP, and C-peptide did not differ in response to 7 d of high versus low daily steps (all *P* > 0.05; see Supplemental Table 1, Supplemental Digital Content, showing serum hormone concentrations before and after the intervention, http://links.lww.com/MSS/C951).

### Precursor pool

After completion of the ^2^H_2_O-dosing protocol (day 1), body water deuterium enrichment reached 0.57% ± 0.07% and 0.56% ± 0.07% in the low and high physical activity groups, respectively. Over the study period, body water enrichment slightly increased and averaged 0.67% ± 0.07% and 0.65% ± 0.07% in the low and high physical activity groups, respectively.

Body water enrichment resulted in a serum-free [^2^H]alanine enrichment of 3.01 ± 0.33 and 2.96 ± 0.38 mole percent excess on the day of surgery (day 8) in the low and high physical activity groups, respectively. No differences between groups were found for body water or serum-free enrichments.

### Tissue protein synthesis rates

**FIGURE 4:**
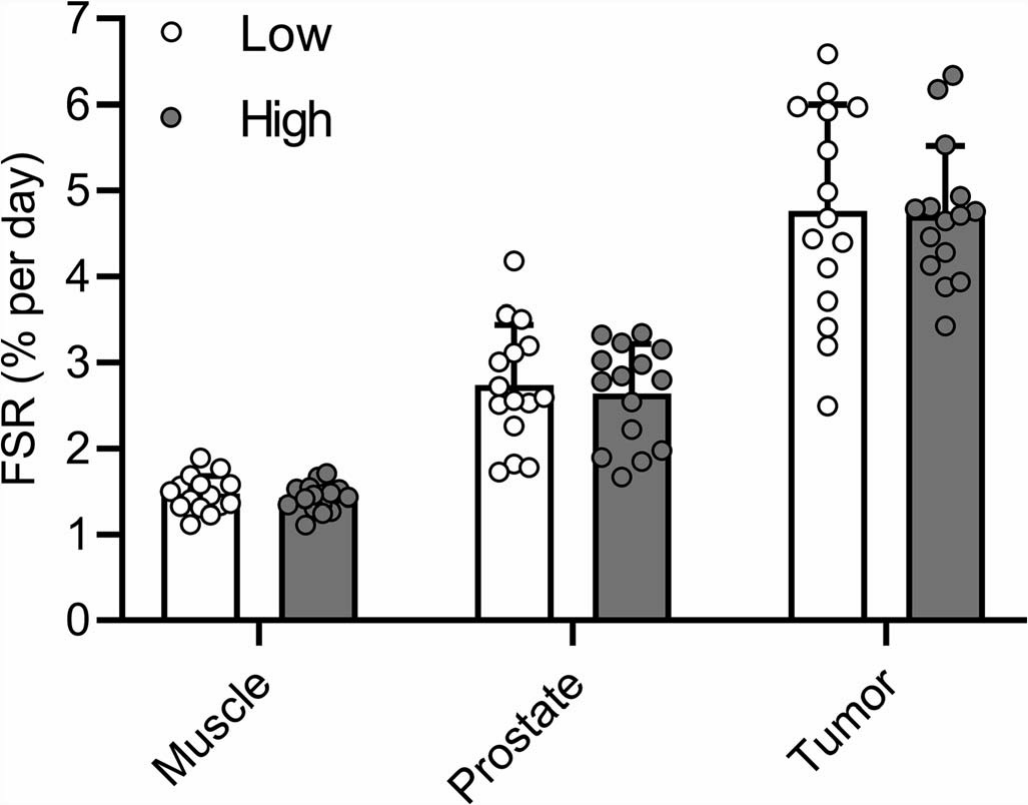
Tissue protein synthesis rates (FSR, %·d^−1^) of vastus lateralis muscle, healthy prostate, and prostate tumor tissue, calculated with body water deuterium enrichments. Tissue-specific protein synthesis rates were compared between groups using independent-samples *t*-tests for vastus lateralis muscle samples, prostate tissue, and prostate tumor tissue. For explorative purposes, FSR values were compared between the different tissues using repeated-measures ANOVA with tissue as within-subject factor and group as between-subject factor. No differences between groups were found. Protein synthesis rates of healthy prostate tissue were 1.9-fold higher than muscle protein synthesis rates (*P* < 0.001). Protein synthesis rates of prostate tumor tissue were 1.9-fold higher than healthy prostate protein synthesis rates (*P* < 0.001) and 3.3-fold higher than muscle protein synthesis rates (*P* < 0.001). Low, low physical activity group; High, high physical activity group.

After protein extraction, tissue protein synthesis rates were assessed (Fig. 4). In vastus lateralis muscle tissue, protein synthesis rates averaged 1.47 ± 0.21 and 1.42 ± 0.16%·d^−1^ in the low and high physical activity groups, respectively. In healthy prostate tissue, protein synthesis rates averaged 2.74 ± 0.70 and 2.64 ± 0.58%·d^−1^, respectively. In prostate tumor tissue, protein synthesis rates averaged 4.76 ± 1.23 and 4.72 ± 0.80%·d^−1^, respectively. Table 4 presents the tissue protein synthesis rates calculated with body water enrichment and with serum-free [^2^H]alanine enrichment as a precursor pool. For both methods, no differences in muscle, healthy prostate, or prostate tumor tissue protein synthesis rates were observed between groups. For explorative purposes, protein synthesis rates of different tissues were compared, only showing a main effect of tissue. Protein synthesis rates in prostate tumor tissue were 1.9 ± 0.6 and 3.3 ± 0.8 times higher than protein synthesis rates in healthy prostate tissue and skeletal muscle tissue, respectively (both *P* < 0.001).

**TABLE 4:** Tissue-specific protein synthesis rates.

		Saliva	Serum	Between-Group Differences (*P* Value)	
	*n*	Mean ± SD	Mean ± SD	Saliva	Serum
Muscle FSR (%·d^−1^)					
Low	15	1.47 ± 0.21	1.21 ± 0.17	0.484	0.379
High	15	1.42 ± 0.16	1.16 ± 0.10		
Total	30	1.45 ± 0.18	1.19 ± 0.14		
Prostate FSR (%·d^−1^)					
Low	15	2.74 ± 0.70	2.25 ± 0.60	0.685	0.623
High	15	2.64 ± 0.58	2.16 ± 0.46		
Total	30	2.69 ± 0.63	2.20 ± 0.52		
Prostate tumor FSR (%·d^−1^)					
Low	15	4.76 ± 1.23	3.89 ± 0.97	0.905	0.885
High	15	4.72 ± 0.80	3.85 ± 0.55		
Total	30	4.74 ± 1.02	3.87 ± 0.77		

Values are mean ± SD. The columns “Saliva” and “Serum” present the tissue-specific protein synthesis rates as calculated with body water enrichment and serum-free [^2^H]alanine enrichment as a precursor pool, respectively.

Low, low physical activity group; High, high physical activity group.

## DISCUSSION

In the present study, we observed that a low or high physical activity level (i.e., <4000 vs >14,000 steps per day during 1 wk in the low and high activity groups, respectively) did not modulate muscle, healthy prostate, or prostate tumor tissue protein synthesis rates. Furthermore, we showed that prostate tumor tissue protein synthesis rates are nearly twofold higher when compared with prostate tissue protein synthesis rates.

After ^2^H_2_O loading, we successfully subjected patients to a low or high daily physical activity level for 7 d before prostate tumor resection. The 4000 steps per day in the present study represent a more sedentary lifestyle, whereas 14,000 steps per day are well above the normal physical activity level reported in both healthy people aged 50–94 yr, who perform between 2000 and 9000 steps per day (42), and prostate cancer patients, who perform between 5500 and 7000 steps per day (43). Furthermore, protein intake in the present study did not differ between the treatment groups and aligned with values observed in studies with healthy older men (44,45), as well as the average values observed in our recent study in patients with (locally) advanced prostate cancer (43). The greater activity level did not result in higher muscle protein synthesis rates. This seems to be in contrast with previous studies, showing robust changes in daily muscle protein synthesis rates after changes in physical activity level (23–25). However, along with methodological differences between the previous studies (i.e., longitudinal vs parallel design, intravenous tracer infusion vs deuterium dosing), differences in physical activity levels were much greater when compared with the present study. Specifically, the previous studies examined the impact of resistance-type exercise training (24% higher muscle protein synthesis rates in exercised compared with nonexercised leg) (23), limb immobilization (36% lower muscle protein synthesis rates in immobilized compared with control leg) (25), or a more severe step reduction protocol to less than <1200 steps per day (27% lower muscle protein synthesis rates during step reduction) (24). In line with the latter study, Breen et al. (46) included healthy older adults who reduced their habitual daily activity from 5962 ± 695 to 1413 ± 110 steps per day, resulting in a 26% decrease of postprandial myofibrillar synthesis rates. It could therefore be speculated that the physical activity level in the low step condition (~3600 steps per day) in the present study was still sufficient to maintain muscle protein synthesis rates.

Like muscle, no differences in protein synthesis rates were found between the low and high activity groups for healthy prostate (2.74 ± 0.70 and 2.64 ± 0.58%·d^−1^, respectively) and prostate tumor (4.76 ± 1.23 and 4.72 ± 0.80%·d^−1^, respectively) tissue. Despite compelling preclinical data on the tumor-growth inhibiting effects of increasing physical activity level, our data indicate that a short-term low or high physical activity level does not directly modulate tumor protein synthesis rates. The possibility exists that more intense exercise applied over a more prolonged period is required to elicit tumor-growth inhibiting effects, by, for example, modulating hormonal/growth factors (17), release of myokines (12), improved immune function (17), and changes in tumor vascularization (18). Furthermore, the potential inhibitory effects of exercise on prostate and prostate tumor protein synthesis rates could have been offset by the accompanying higher daily energy intake in the high versus the low physical activity group. However, this is speculative as we are still unaware of clinical evidence of the impact of energy intake and physical activity patterns on protein balance in healthy organ tissue and tumor tissues.

Despite the fact that performing a short-term high or low number of daily steps does not seem to modulate prostate or prostate tumor protein synthesis rates, our findings provide novel insights into prostate and prostate tumor metabolism. As far as we know, there are no data available on prostate and/or prostate tumor protein synthesis rates *in vivo.* Although the higher protein synthesis rates in prostate tumor versus healthy prostate tissue could suggest that there is net tumor tissue accretion, this is not *per se* the case. Tissue protein accretion is determined by the dynamic balance between tissue protein synthesis and breakdown rates. Interestingly, findings from other studies show that tumor protein synthesis rates are not consistently higher than protein synthesis rates of the organ tissue in which the tumor is embedded. For example, we have previously reported lower protein synthesis rates in pancreatic tumor tissue when compared with healthy pancreatic tissue (22). Studies in gastrointestinal and colorectal cancers, however, show higher FSRs in the cancerous tissue compared with the corresponding healthy tissue (47,48). As far as we know, this is the first study to assess prostate and prostate tumor protein synthesis rates *in vivo* in humans.

The apparent differences in tissue protein synthesis rates between healthy prostate and prostate tumor may provide us with indications for preferred intervention strategies to reduce tumor tissue accretion, for example, to focus on the molecular pathways of tumor tissue synthesis or breakdown. For example, the higher protein synthesis rates in prostate tumor than in healthy prostate tissue could indicate that interventions (like physical activity, nutritional or pharmacological interventions) should focus on inhibiting tumor protein synthesis. This directly highlights the potential of the applied ^2^H_2_O methodology, which enables us to assess protein synthesis rates in cancerous and noncancerous tissue, as well as the effect of several interventions on tumor protein synthesis rates in patients under free-living conditions.

There is ongoing discussion about the impact of physical activity level on tumor metabolism. However, as we did not measure baseline physical activity levels, our data do not allow for a conclusion to be drawn over the effect of individual changes in habitual physical activity level on tumor protein synthesis rates. Randomized controlled trials evaluating greater changes in the intensity of daily physical activity (e.g., greater walking cadence, [49]) and/or supervised exercise training (endurance and/or strength training) should be performed to confirm or contrast our present findings. A second limitation is the absence of habitual dietary intake data, limiting the opportunity to check for possible confounding factors induced by changes in dietary intake.

## CONCLUSIONS

In conclusion, a short-term high or low physical activity level does not influence prostate tumor protein synthesis rates. Studies on the efficacy of short- and long-term exercise interventions to modulate tumor protein synthesis rates and attenuate tumor progression are warranted to understand the impact of lifestyle as an adjuvant therapy in the prevention and treatment of cancer.
